# Mechanism of bacteriophage-induced vaterite formation

**DOI:** 10.1038/s41598-024-71638-2

**Published:** 2024-09-03

**Authors:** Andrzej Borkowski, Paweł Działak, Katarzyna Berent, Marta Gajewska, Marcin D. Syczewski, Mirosław Słowakiewicz

**Affiliations:** 1 Faculty of Geology, Geophysics and Environmental Protection, AGH University of Krakow, Al. Mickiewicza 30, 30-059 Kraków, Poland; 2 Academic Centre for Materials and Nanotechnology, AGH University of Krakow, Al. Mickiewicza 30, 30-059 Kraków, Poland; 3 GFZ German Research Centre for Geosciences, 14473 Telegrafenberg, Potsdam, Germany; 4 Faculty of Geology, University of Warsaw, Ul. Żwirki i Wigury 93, 02-089 Warsaw, Poland

**Keywords:** Amorphous calcium carbonate, Vaterite, Calcite, Bacteriophage, Viruses, Precipitation of carbonates, Biogeochemistry, Mineralogy, Nanobiotechnology

## Abstract

This study shows how bacterial viruses (bacteriophages, phages) interact with calcium carbonate during precipitation from aqueous solution. Using electron microscopy, epifluorescence microscopy, X-ray diffraction, and image analysis, we demonstrate that bacteriophages can strongly influence the formation of the vaterite phase. Importantly, bacteriophages may selectively bind both amorphous calcium carbonate (ACC) and vaterite, and indirectly affect the formation of structural defects in calcite crystallites. Consequently, the surface properties of calcium carbonate phases precipitating in the presence of viruses may exhibit different characteristics. These findings may have significant implications in determining the role of bacterial viruses in modern microbially-rich carbonate sedimentary environments, as well as in biomedical technologies. Finally, the phage-vaterite system, as a biocompatible material, may serve as a basis for the development of promising drug delivery carriers.

## Introduction

Calcium carbonate (CaCO_3_) is a mineral that exists in six polymorphic forms, namely calcite, vaterite, aragonite, ikaite, monohydrocalcite and calcium carbonate hemihydrate. Calcite (trigonal), the most stable polymorph, is widespread in the Earth’s crust whereas aragonite (orthorhombic) is present in e.g. nacre and various mollusc shell, lime mud and ooids. The least stable polymorphs of CaCO_3_, vaterite (hexagonal), and ikaite (monoclinic), can be found in fish otoliths^1^ and in mineral springs^2^; and ikaite is forming only in very cold water (− 3 to + 3 °C). The crystals of these four polymorphs have distinct shapes i.e., calcite is rhombohedral, aragonite is rod-like, needles and fibres, vaterite is spherical and ikaite is spindle-shaped. In general, the size and morphology of calcium carbonate may be affected by various factors including agitation time^3^, temperature of the synthesis^3^ and presence of additional chemical compounds like polyethylene glycol^4^ and sodium dodecyl sulphate^5^.

The mechanism of vaterite formation consists of several steps. Initially, amorphous calcium carbonate (ACC) forms in the solution. Subsequently, ACC undergoes dehydration, and spherical vaterite begins to form. When all ACC is exhausted, the vaterite spherules continue to grow via Ostwald ripening. Finally, the vaterite can be transformed into calcite^6^ or vaterite can be stabilised and not transformed into calcite^7–9^.

To look deeper into these crystallisation phenomena, Słowakiewicz et al.^10^ suggested that bacteriophages may also induce vaterite formation. However, the mechanism of vaterite formation in the presence of bacteriophages still remains unknown. It was later suggested that bacteriophages may behave like crystallisation seeds or nucleation sites^11,12^ and thus somehow could stabilise the recrystallisation of vaterite to calcite. Moreover, bacteriophages were successfully used as agents that modify the surface of various minerals e.g., carbonates^10^, iron oxides^11^, iron sulphides^12^, copper sulphide^13^ or even silicious material^14,15^. Therefore, it appears that viruses may also have geological implications in various environments, where they can take part in virus-mediated mineralisation and in biofilm formation^16–22^.

In addition, vaterite seems to be the most interesting from the point of view of biotechnology because of its useful properties including high porosity, biocompatibility and higher solubility in water^23^. Hence, porous vaterite particles can be used as a bed for adsorption of chemical compounds^23^. What is more, vaterite has been extensively studied as a drug carrier^24–26^, but it has never been combined with bacteriophages. Despite the lack of strong evidence regarding the role of bacteriophages in virus-mediated mineral precipitation, they may be either integrated into the mineral structure or somehow attached to the surface. While in the case of bacteria it has been shown that they can participate in biological mineralisation processes^27^, the possible role of viruses (particularly bacteriophages) is still poorly understood. This is important because in nature, viruses are usually much more abundant than bacterial cells and their size and the often crystalline nature of their capsids may suggest a possible role for them in the environment, where calcium carbonate and also other minerals can precipitate. Therefore, this paper presents the results of laboratory studies aimed at identifying the location of bacteriophage incorporation within the calcium carbonate mineral phases and the fate of bacteriophages during mineral phase transformation. This study may be promising for further studies on vaterite-bacteriophage as carriers for targeted phage-therapy. Importantly, it can help explain to the origin of nanospheres in microbially-dominated carbonate systems first reported by Folk^28^ and this could become a game-changer in basic concepts of carbonate sedimentology.

## Materials and methods

### Preparation of bacteriophage suspensions

Two different bacteriophages were selected for the study, which differ primarily in the presence of a lipid membrane around the capsid*.* Non-enveloped *Escherichia* virus P1 (DSMZ, DSM 5757) and lipid enveloped *Pseudomonas* virus Phi6 (DSMZ, DSM 21518), were cultured using a modified double-agar layer method^29^. The protocol has been previously tested and described^11,12^. Briefly, three media were prepared: bottom agar—tryptic soy broth (TSB) containing 1.5% agar, top agar—TSB containing 0.75% agar, and liquid TSB without agar. The media were sterilised at 121 °C for 30 min and cooled down to 50 °C. Bottom agar was poured onto the Petri dishes, and plates were dried to remove the condensed vapour on the surface of the solid medium. Sterile 1 M MgSO_4_ solution was added to the cooled liquid TSB, and the final concentration was set at 5 mM. To a 15 mL tube with 0.1 mL of diluted bacteriophage solution, 1 mL of liquid TSB containing 5 mM MgSO_4_ and 0.1 mL of an overnight (18 h) liquid bacterial culture was added (OD550 ranges for *Escherichia coli* and *Pseudomonas syringae* were 0.7–0.9 and 0.4–0.5, respectively). Cooled top agar (4 mL) containing 5 mM MgSO_4_ was added to the bacterial solution. The tube was gently mixed, and the solution was immediately poured onto a bottom agar plate. Solidified plates were incubated for 24 h at 37 °C and 48 h at 25 °C for the *E. coli* and *P. syringe*, respectively. Next, a top-agar layer was placed in a 50 mL polypropylene tube and vigorously shaken with Tris-MgCl_2_ buffer. Then, the tube was centrifuged (4400 × g) to remove all the residues of agar and bacteria. Subsequently, the supernatants were transferred to 2 mL polypropylene tubes and centrifuged (24,250 × g). The supernatants were completely discarded, and viral pellets were resuspended in 0.9% NaCl solution and filtered using a syringe filter (RC, 0.22 µm). The bacteriophage yield was set at 1 × 10^11^ virions mL^−1^.

### Scheme of experiments

**Fig. 1:**
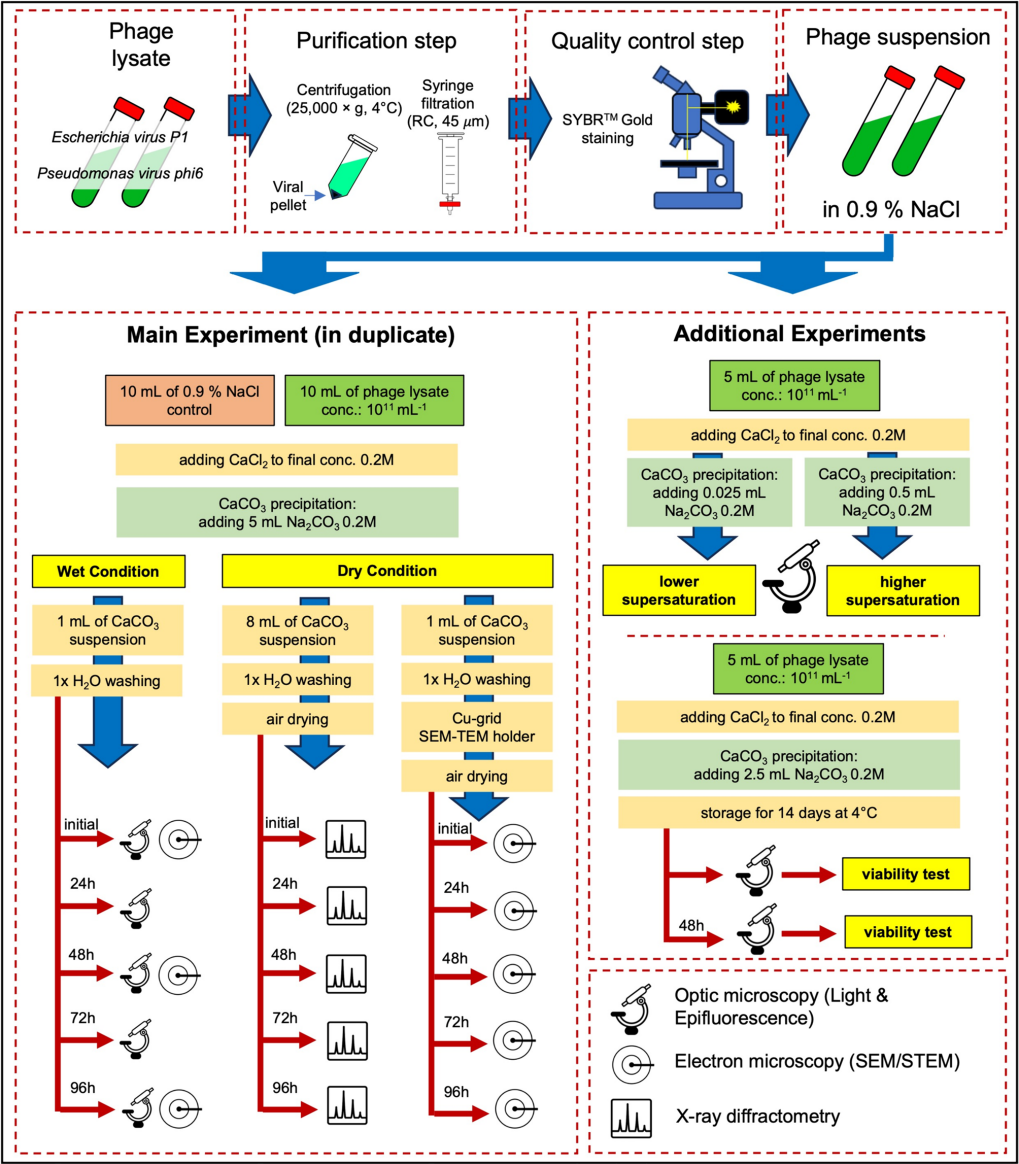
Scheme of conducted experiments with phages and calcium carbonate precipitations.

The general scheme of experiments is presented in Fig. 1. Previously obtained purified phage lysates were examined for the presence of bacteria and bacterial debris. For this purpose, staining with SybrGold was applied, followed by observations under an epifluorescence microscope (blue filter, DM500 with a band-pass 460–490 nm excitation filter). Any bacterial residues and bacterial cells would be clearly visible in phage suspensions after purification using this method. Phage suspensions that passed the quality control stage (lack of bacterial debris) were used in precipitation experiments. These experiments were divided into two stages: Main Experiment and Additional Experiments. The Main Experiment was conducted in two independent repetitions at a phage concentration 10^11^ mL^−1^. It consisted of two variants: Wet Condition and Dry Condition. The difference lay in the fact that in the first variant, precipitated sediments were rinsed in deionized water and then left in the aqueous suspension. In the second variant, obtained sediments were rinsed in deionized water, dried, and then, as air-dried sediments, were examined over subsequent days according to the presented scheme (Fig. 1). For the Main Experiments, the carbonate precipitation protocol was as follows. A specific volume (10 mL in clean 25 mL glass containers at a temperature of 22 °C) of phage suspension in 0.9% NaCl was enriched with calcium chloride (anhydrous CaCl_2_, Sigma-Aldrich) to a final concentration of 0.2 M. The control sample was a 0.9% NaCl aqueous solution without phages, enriched with CaCl_2_. Subsequently, 5 mL of sodium carbonate solution (0.2 M, based on anhydrous Na_2_CO_3_, Sigma-Aldrich) was added to the prepared solutions at once. During calcium carbonate precipitation, the solutions were not mixed. Only after adding sodium carbonate the solutions were gently mixed by one or two tube inversions and left for 15 min. After this time, the obtained precipitates were treated according to the experiment's variant scheme (Fig. 1). During the experiment conducted under wet conditions, crystallites remaining in the aqueous suspension were examined every 24 h using a fluorescence microscope. This allowed for the evaluation of both the changes in the crystallites’ shape and their binding with viruses using SybrGold staining.

### Additional experiment I: precipitation at different supersaturation states

The precipitation of calcium carbonate in the presence of phages was also carried out, considering the state of supersaturation relative to vaterite. Supersaturation was defined as^30^:

$$S = \sqrt {\frac{{a_{{{\text{Ca}}^{2 + } }} \times a_{{{\text{CO}}_{3}^{2 - } }} }}{{K_{sp} }}} ,$$

where *S*—supersaturation state, $a_{{ca^{2 + } }}$ and $a_{{CO_{3}^{2 - } }}$ are activities of calcium and carbonate ions, *K*_*sp*_—solubility product of vaterite (1.22 × 10^−8^). The approximation of ion activity was based on molar concentrations. The experiment was conducted in two variants: (i) a lower supersaturation state variant (lower S = 2.8 × 10^2^) where 25 µL of Na_2_CO_3_ solution was added to 5 mL of phage-enriched CaCl_2_ suspension, and (ii) a higher supersaturation state variant (higher S = 8.2 × 10^2^) where 500 µL of Na_2_CO_3_ solution was added to 5 mL of phage-enriched CaCl_2_ suspension. The initial concentrations of reagents were the same as in the Main Experiment. After precipitation, the calcium carbonate suspension was washed, stained with SybrGold, and examined under an epifluorescence microscope (blue filter, DM500 with a band-pass 460–490 nm excitation filter).

### Additional experiment II: viability of viruses after precipitation

An experiment was conducted to investigate whether phages, after the precipitation of calcium carbonate and subsequent recrystallisation to calcite, could retain viability, understood as the ability to infect. The sedimentation experiment was carried out a similar fashion to the Main Experiment, followed by washing the carbonate suspension with deionized water and air-drying. Subsequently, the carbonate sediment was stored for 14 days at 4 °C. Afterwards, the sediment was reintroduced into 0.9% NaCl, and the presence of infective phages was examined using the standard lysate method on a semi-solid agar. After 48 h, when the recrystallisation process to calcite was visible, the presence of phages was re-examined using the lysate method.

### Electron microscopy and selected area electron diffraction

Morphological observations of aqueous suspensions were performed using an FEI Versa 3D scanning electron microscope (SEM). Scanning transmission electron microscopy (STEM) imaging was conducted using FEI's STEM II angularly resolved electron detector. SEM measurements were done at an accelerating voltage of 5 kV and 10 kV. Transmission electron microscopy (TEM) investigations were carried out on a Tecnai TF20 X-TWIN (FEG) microscope (FEI), operating at an accelerating voltage of 200 kV. Bright field (BF) and high resolution (HR) imaging, as well as selected area electron diffraction (SAED) were applied. Samples for SEM and TEM observations were prepared by drop casting of water suspensions on the carbon-coated copper TEM grids. All microscopic images that were collected during experiments are deposited in the repository due to their high volume (10.5281/zenodo.11159734). Here, only the most important representative SEM, TEM and epifluorescence microscope images are shown.

### XRD

X-ray diffraction (XRD) data were determined using a Rigaku SmartLab diffractometer, with a graphite monochromator and rotating on a copper anode. The data acquisition was in the range of 5–75° 2θ with a 0.05° gradation and a counting time of 1 s per grade. For diagnostic purposes, during subsequent days of measurements, diffractograms were recorded in the range of 20–35° 2θ, encompassing well-distinguishable peaks for calcite and vaterite.

## Results

### Association of viruses with vaterite

**Fig. 2:**
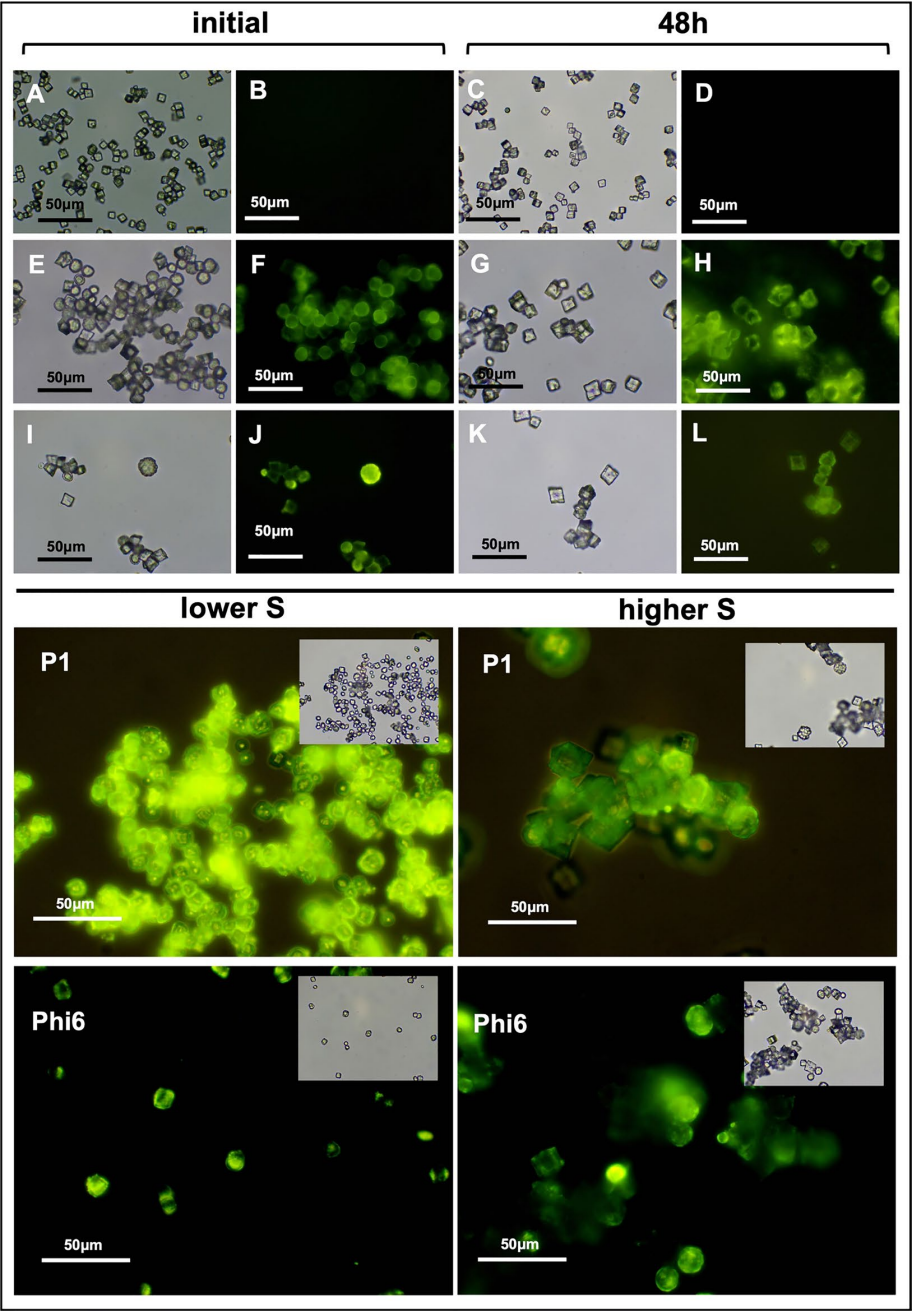
Epifluorescence microscope. Crystallites after precipitation (initial) and after 48 h under wet conditions: (**A**–**D**) control; (**E**–**H**) P1; (**I**–**L**) Phi6. Binding of viruses with vaterite crystallites at different supersaturation conditions (low S and high S). Green fluorescence comes from viruses associated with carbonate particles.

The green fluorescence originated from the viral particles that were associated with the crystallites (Fig. 2). Immediately following precipitation, significant differences were observed between the control samples and samples with P1 and Phi6 (Fig. 2A,E,I, respectively). The crystallites in the control samples appeared relatively homogeneous (about 8–10 μm in diameter) and were evenly dispersed in the microscopic image, with no visible fluorescence (Fig. 2B). Crystallites precipitated in the presence of P1 and Phi6 bacteriophages were of two types. Both rhombohedral crystals (calcite) (about 10 μm in diameter) and spherical crystallites of various diameters (about 5–15 μm in diameter) (vaterite) were observed. Notably, bright fluorescence was primarily exhibited by the spherical structures, while the precipitated calcite crystallites were much less visible under the fluorescence microscope (Fig. 2F,J). The very bright fluorescence may indicate significant packing of viral particles within the precipitated spherical vaterite structures. Additionally, the maximum size of the crystallites precipitated in the presence of viruses, was approximately twice as large (max. 20 μm in diameter).

During the experiment conducted under wet conditions, significant changes in the shape of the crystallites were noticed after 24 h. In particular, spherical structures seemed to disappear, and calcite-structured crystallites began to form. After 48 h, the spherical structures had almost completely disappeared (Fig. 2G,K). Moreover, a very clear glow was visible around the visible rhombohedral calcite crystallites, likely emanating from the released viral particles (Fig. 2H,L). No differences were noted for the control experiment (Fig. 2C,D). In the following days (up to 96 h), the image remained essentially unchanged, with rhombohedral crystallites predominating.

Selective binding of viruses to spherical structures became even more apparent when calcium carbonate was precipitated at different saturation states (Fig. 2). At a lower saturation state (Fig. 2: lower S), mainly spherical structures (size) with a strong fluorescence were formed, with all visible crystallites showing fluorescence. At a higher saturation state (Fig. 2: higher S), both spherical structures (showing strong fluorescence) and rhombohedral crystallites with weaker or no fluorescence were visible. This was true for both P1 and Phi6 bacteriophages. It should be noted that the fluorescence observed with the larger spherical structures was not homogeneous. This was particularly evident with Phi6 (Fig. 2: higher S, Phi6). However, it is not possible to explain unequivocally what these differences result from. It could be either an effect of the morphology of the calcium carbonate particles in the surroundings of viruses or an uneven distribution of viral particles.

### Morphology of precipitates

**Fig. 3:**
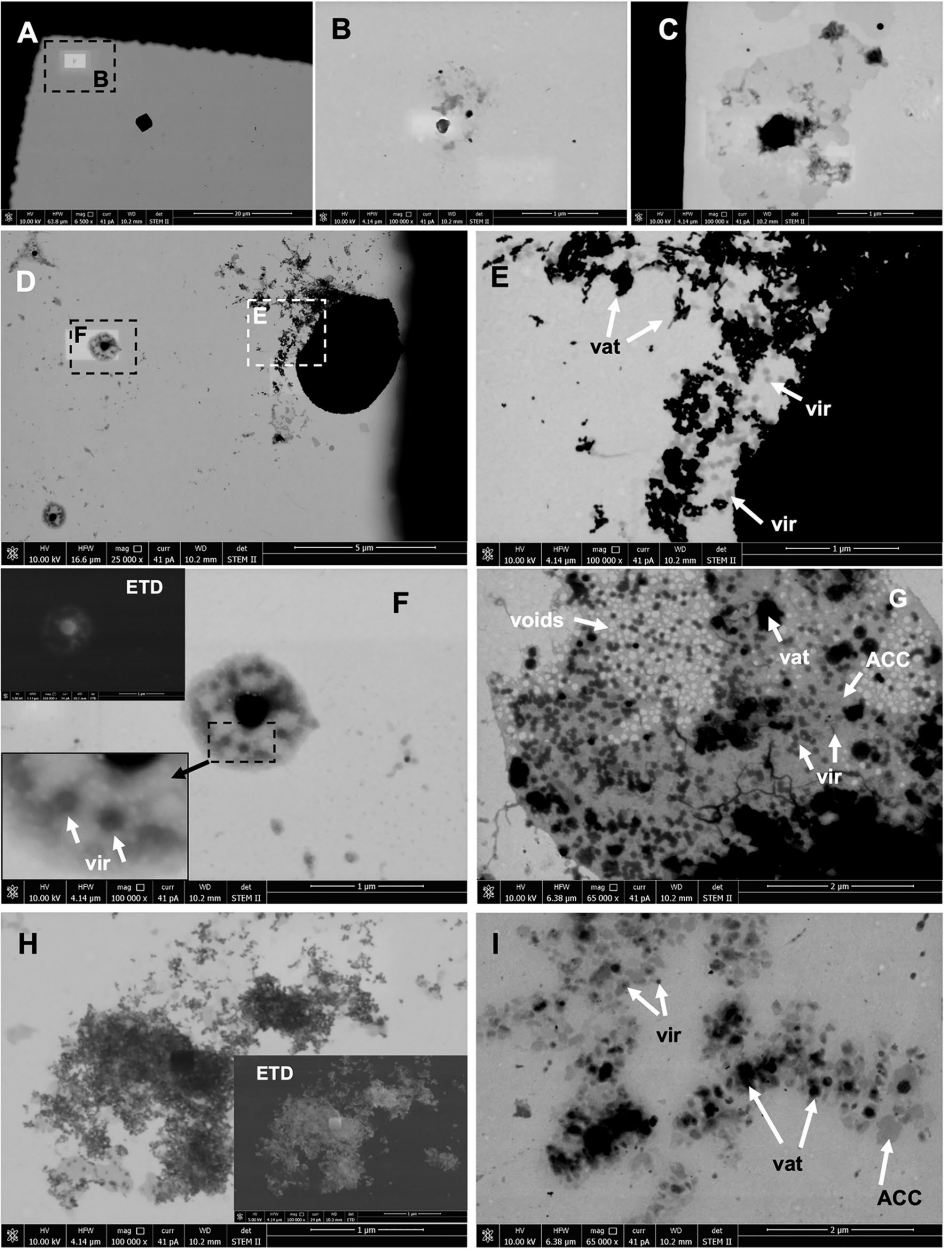
STEM/SEM images directly after precipitation (initial for both experiments under wet and dry conditions). (**A**–**C**) Control; (**D**–**G**) precipitates with P1 phages; (**H**, **I**) precipitates with Phi6 phages. Images show possible VLPs (vir), vaterite particles (vat) and ACC. The insets (**F**, **H**) show a magnification of an analysed structure with possible phage capsids and images of the same area under the Everhardt-Thornley detector (ETD) for secondary electrons (SE).

Optical microscopy observations will not provide answers as to whether viruses are bound to the surface of the precipitates (in the sense of adsorption) or perhaps encapsulated within the precipitated structures. More conclusive results were revealed upon examination of the precipitates using STEM immediately after precipitation (Fig. 3). In the control, a rather homogeneous picture was recorded. The presence of crystallites with a regular structure was observed (Fig. 3A), as well as the presence of less regular, very fine structures, albeit fewer in number (Fig. 3B). The presence of irregularly structured, electron-rare structures (in shades of grey) was also noted (Fig. 3C). However, these structures are not necessarily identified with ACC. They may also be incidental contaminants, especially as they occur very rarely in the control material.

With phages, much more morphological diversity was revealed. Both spherical structures, likely vaterite, consisting of very fine electron-dense nanocrystallites were observed, among which virus-like particles (VLPs) of about 50–100 nm in size are also visible (Fig. 3D,E). This can be confirmed because it was possible to record the slightly damaged spherical structure of vaterite spheroids.

In addition, for P1, the presence of smaller complex structures (about 1000 nm) was noted, in which a central electron-dense crystallite surrounded by viral particles was clearly visible (Fig. 3D,F). The viral particles were likely surrounded by ACC. The experiment with P1 also revealed the presence of structures of a larger size, albeit similarly structured (Fig. 3G). Both electron-dense areas and particles with sizes corresponding to the P1 viral capsids (50–100 nm) were visible, as well as areas resembling voids after the release of viral capsids. It should be noted that VLPs in the STEM image were characterised by different electron densities.

A slightly different image was obtained with Phi6. Here, in addition to similar larger spherical vaterite structures, many very fine electron-dense nanocrystallites were noted, also surrounded by grey matter (Fig. 3H). Moreover, both irregular crystallites and more VLPs were visible. This is well visualized in Fig. 3I, where both ACC and vaterite crystallites and VLPs are likely visible.

**Fig. 4:**
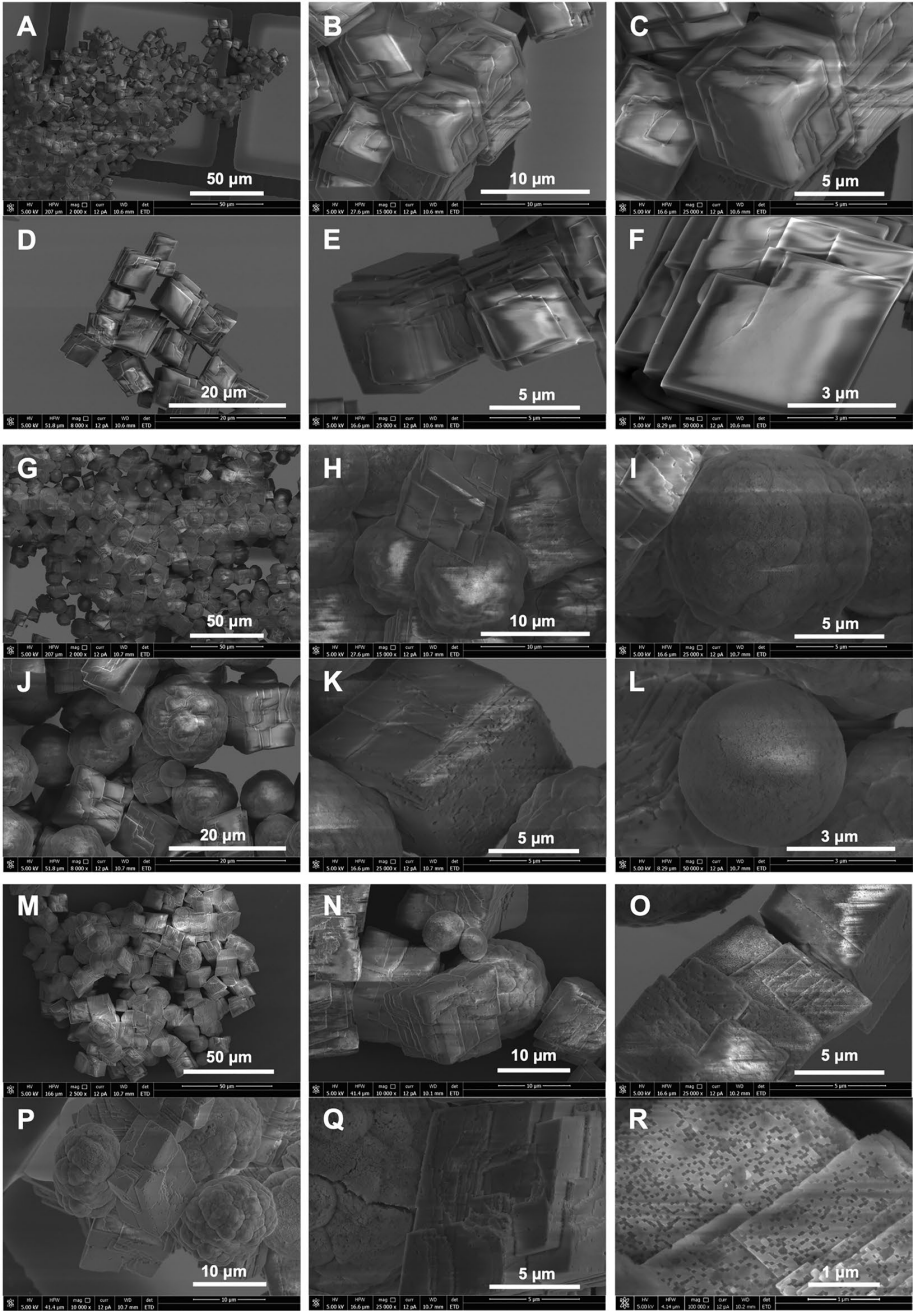
SEM images directly after precipitation (initial for experiments under wet and dry conditions). (**A**–**F**) Control; (**G**–**L**) precipitates with P1 phages; (**M**–**R**) precipitates with Phi6 phages.

In contrast, SEM analysis revealed significant differences in the morphology of the crystal structures (Fig. 4). In the SEM image, well-developed rhombohedral crystal structures with smooth surfaces were visible in the control experiment (Fig. 4A–F). The size of the crystallites was about 5–8 μm. No spheroidal or irregular structures were noted. No porous structures were observed on the surface of the crystalline forms. Only multi-face crystal forms were visible.

A different picture emerged from experiments with P1 (Fig. 4G–L). Here, rhombohedral forms were also visible, although they differed in surface structure from the control (Fig. 4K). The surfaces were often uneven and rough, often with the presence of small cavities and defects. The size of these crystallites was also slightly larger than in the control, reaching around 10 μm. However, the most significant difference was the presence of spheroidal structures that can be identified as vaterite (Fig. 4G–J,L). They varied in size from 4 to 15 μm. These structures had a spheroidal form (Fig. 4L), but there were also “cauliflower-like” forms (Fig. 4I,J).

The surface was rough with a porous structure, making it impossible to distinguish between vaterite crystallites and viral particles on the surface of these structures. A similar image was recorded in an experiment with Phi6 (Fig. 4M–R). However, the sizes of the rhombohedral crystalline structures were slightly larger (more than 10 μm). It is also important to note that surfaces with numerous defects, resembling voids with sizes similar to viral particles, were also found (Fig. 4R). Similarly, the spheroidal structures could reach larger sizes (< 20 μm; Fig. 4N). In addition, it seems that the “cauliflower-like” structures are predominant in this case (Fig. 4P). In contrast, as with P1, the surface was heterogeneous and seemingly porous. It was not possible to distinguish between VLPs and crystallites building spheroidal structures.

### Recrystallisation under wet conditions

**Fig. 5:**
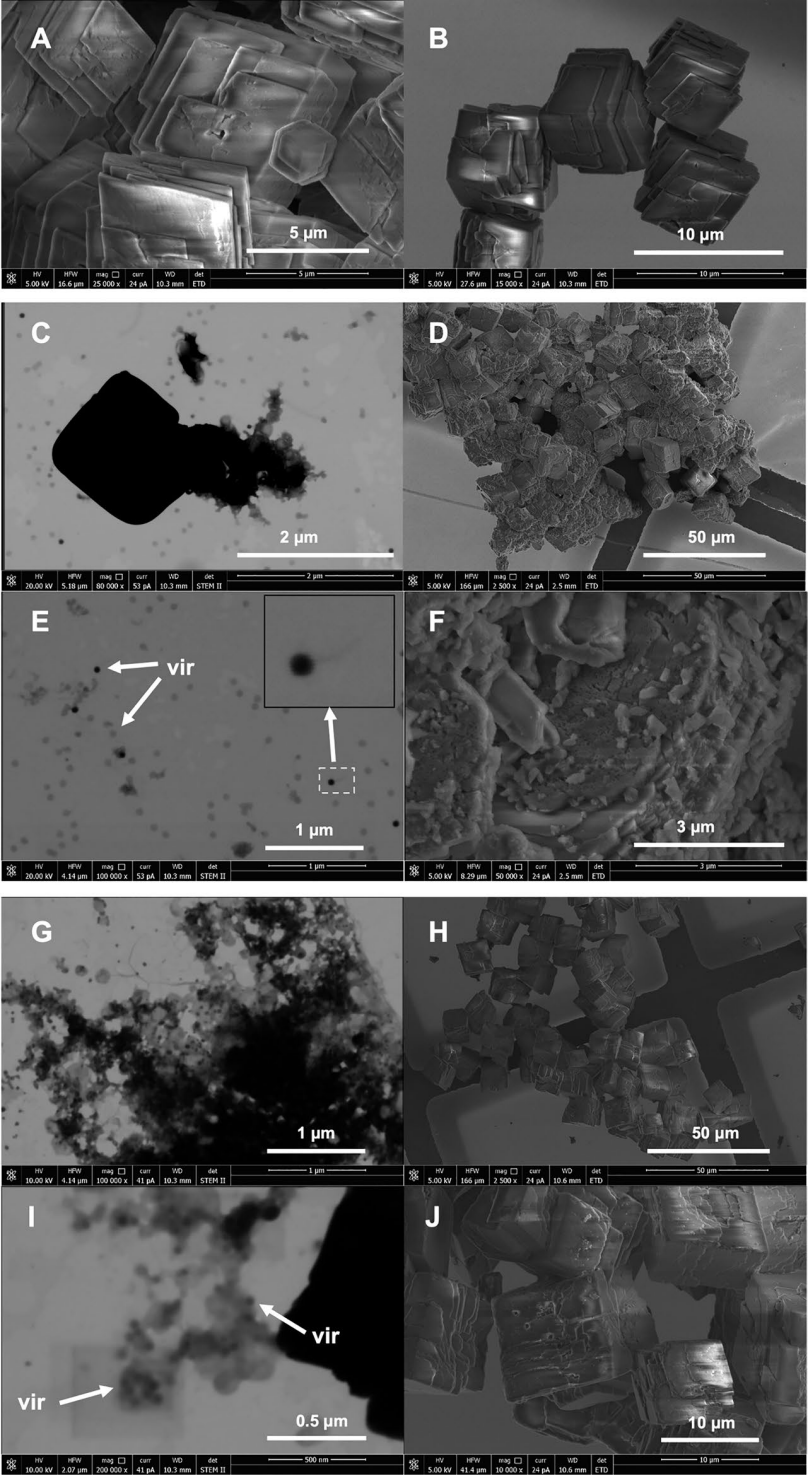
SEM and STEM/SEM images of carbonate precipitates after 48 h (wet conditions). (**A**, **B**) Control; (**C**–**F**) precipitates with P1 phages; (**G**–**J**) precipitates with Phi6 phages. In (**E** and **I**) phages are marked (vir). The inset (**E**) shows a magnification of a single capsid with a visible tail.

The disappearance of spheroidal structures in the aqueous suspension was visualized within 48 h. Epifluorescence microscopy simultaneously revealed the appearance of strong fluorescence around the crystallites remaining in the suspension. In the STEM/SEM image after 48 h, changes in the morphology of the structures and their surroundings could be seen very clearly (Fig. 5). Naturally, in the control, no changes from the initial conditions were noted (Fig. 5A,B). Very similar rhombohedral crystal forms were visible immediately after precipitation.

In contrast, with P1 phages, it was possible to observe the disintegration of the spheroidal forms leading to the formation of quasi-fractal structures (Fig. 5C). Many VLPs or even viruses themselves, can be seen in the surroundings due to the highly visible structure characteristic of P1 (Fig. 5E).

It should be emphasized that VLPs are characterized by varying electron densities. No spheroidal structures were noted in SEM. Only irregular agglomerates of fine crystallites and rhombohedral crystalline structures with the presence of fine crystallites on the surface were visible (Fig. 5D,F). Similarly, with Phi6 phages (Fig. 5G–J), disintegrating spheroidal structures with visible VLPs are also visible in STEM/SEM (Fig. 5G). However, they appear to have been more associated with an electron-rare amorphous substance, possibly ACC (Fig. 5I). The disintegration image of the spheroidal forms took on a massive form and also resulted in the release of a large number of VLPs into the environment. However, it cannot be conclusively shown that the released particles were in fact viruses. The morphology of Phi6 is not as unambiguous in STEM/SEM imaging as in P1. Given the conditions of the experiment, the size of VLPs, their electron permeability, and the similarity with P1 phages, it can be assumed that the visible particles were capsids of Phi6. On the other hand, virtually only rhombohedral crystalline structures with no visible irregular agglomerates were observed in SEM. No significant number of fine crystallites on the surface was noticed either. However, as with P1, the surface of the rhombohedral forms was uneven, jagged, and with visible defects (Fig. 5J).

**Fig. 6:**
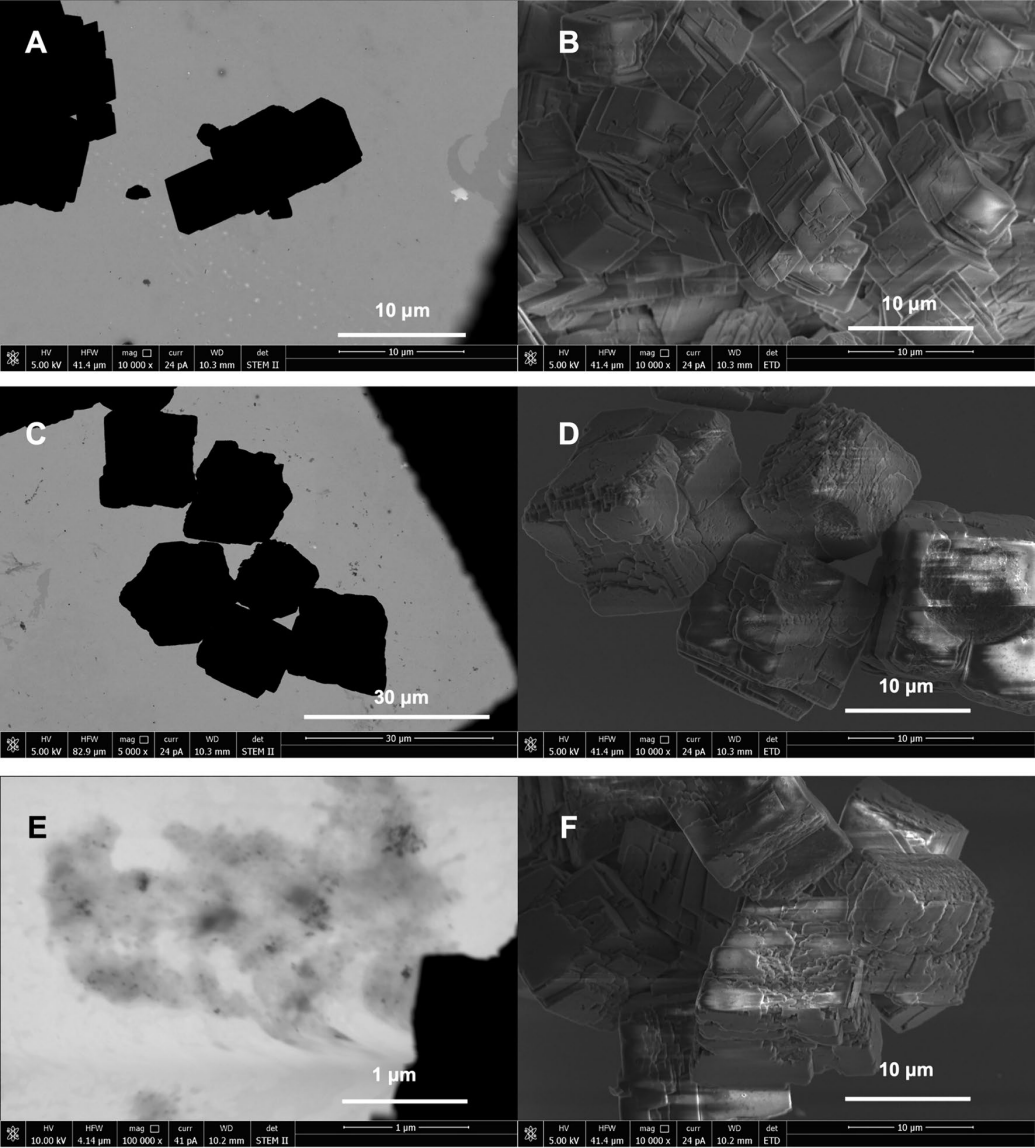
SEM (**B**, **D**, **F**) and STEM (**A**, **C**, **E**) of carbonate precipitates after 96 h (wet conditions) from control, P1 and Phi6 experiment respectively.

The final observations (after 96 h) with electron microscopy also revealed a more uniform image (Fig. 6). In controls, visible crystallites did not reveal any morphological changes (Fig. 6A,B). Even-faces forms were visible, which is well illustrated in STEM/SEM (Fig. 6A). The general conformation of the crystalline forms did not differ from those found at the beginning of the experiment and after 48 h.

With P1, large rhombohedral crystal forms with an uneven surface were visible in STEM/SEM (Fig. 6C). There was a decrease in the number of visible small crystallites and aggregates of viral and mineral particles, which were observed around the rhombohedral forms after 48 h. SEM image shows rhombohedral forms with an uneven surface with numerous defects, but no longer without the presence of fine crystalline forms. The voids of the spheroidal vaterite forms were clearly visible (Fig. 6D). The edges of the rhombohedral forms were not as sharp as in the case of the forms seen in the control.

With Phi6, only rhombohedral forms were also visualized, with a very uneven surface with numerous defects (Fig. 6F). The surface structure of these crystallites appeared porous compared to the control, where only forms with smooth surfaces were visible. Surrounding the rhombohedral forms, an amorphous structure with visible very fine particles somewhat resembling the nanocrystallites from the beginning of the experiment and possible viral particles could additionally be seen (Fig. 6E).

### Stability of vaterite at dry conditions

**Fig. 7:**
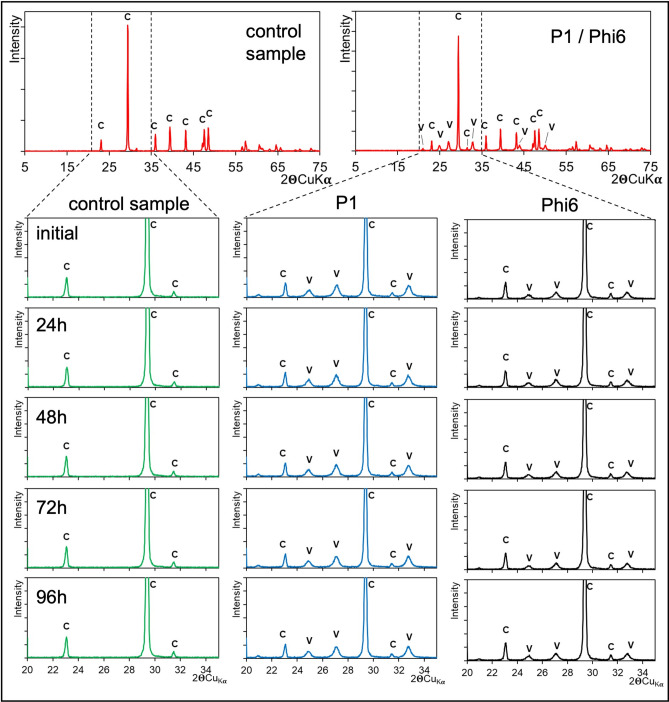
Diffractograms of precipitates measured within 5 days under dry conditions. After precipitation in the presence of P1 and Phi6, calcite and vaterite were noted. After 96 h no changes were found.

Under wet experimental conditions, signs of recrystallisation of precipitates were easily observed. However, the question arises whether any transformations occurred in the precipitates under dry conditions. Over 96 h of XRD measurements, no changes were observed in the XRD pattern for both control and in the presence of P1 and Phi6 (Fig. 7). In the control, only calcite was detected (in both experimental replicates). In samples precipitated in the presence of viruses, both calcite and vaterite were detected (also in all experimental replicates). It appears that with Phi6, there may have been slightly less vaterite compared to samples with P1. However, this could only be assessed semi-quantitatively by comparing the ratios of signals from calcite and vaterite.

### TEM and SAED

**Fig. 8:**
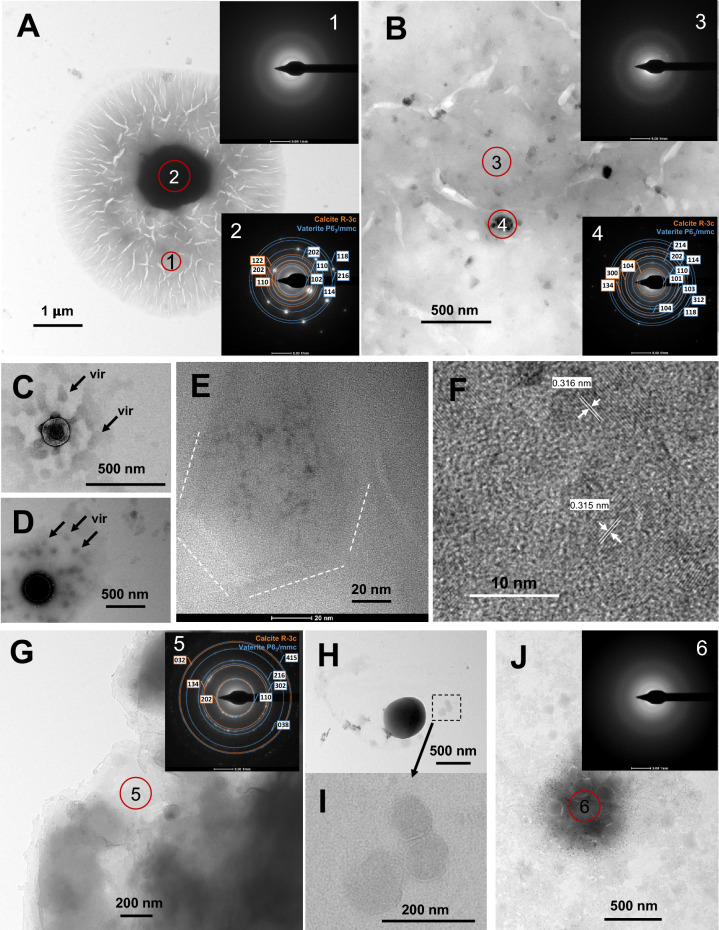
TEM images with SAED patterns directly after precipitation (initial for both experiments under wet and dry conditions). (**A**–**F**) Precipitates with P1 phages; (**G**–**I**) precipitates with Phi6 phages. (**J**) TEM image with SAED pattern after 48 h under wet conditions. Possible VLPs (vir) are marked. The insets show SAED analyses of marked area (numbers 1–6).

In STEM observations, the presence of electron-dense crystallites and nanoparticles, as well as electron-sparse material with an amorphous appearance, was observed. The interpretation of the visible structures suggested the presence of both crystalline and amorphous phases. However, evidence of the presence of phases of such character can only be provided by diffraction studies (e.g., SAED). TEM allowed for the observation of more details of the discussed structures (Fig. 8). The characteristic structures visible in Fig. 3F indeed appear to consist of components—a central phase with a crystalline character and surrounding phases with an amorphous character in which clearly visible phage capsids are embedded (Fig. 8A,C,D). The presence of an amorphous substance, which could be interpreted as ACC, was not limited solely to these structures. SAED analyses confirmed its presence also in the form of irregular clusters along with phases of a polycrystalline character (Fig. 8B). In experiments involving carbonate precipitation in the presence of P1, the presence of capsids with varying electron density could be observed (as shown in Fig. 5E). These phages are easily recognizable under TEM due to their angular shape (Fig. 8E). However, could the electron density result from the precipitation of calcium carbonate on the capsid surface? In the HRTEM image, the presence of structures with evidently crystalline nature can be discerned, but whether this material is precipitated on the capsid surface is unclear (Fig. 8E,F). With Phi6, the presence of an amorphous mineral phase as well as small crystalline structures, often of a polycrystalline nature, could also be demonstrated (Fig. 8G). Similar shape structures composed of a crystalline phase surrounded by an amorphous phase with “embedded” probable Phi6 phage capsids could also be observed (Fig. 8H,I). However, the image of these phages is less clear-cut than in the case of P1. As demonstrated, under wet experimental conditions, after 48 h, there was almost complete disintegration of vaterite spheres and recrystallisation. In addition to typical calcite crystallites, the presence of amorphous phases along with polycrystalline structures and possible phage capsids could also be observed (Fig. 8J).

The SAED analysis allows the identification of the degree of crystallinity of the precipitates and whether amorphous. Based on the result, the mineral phases precipitated in the surroundings of the bacteriophages were determined (Fig. 8, insets 2, 4, and 5). It should be emphasised, however, that these findings are only preliminary due to insufficient data and possible inaccuracy in the analysis. Nevertheless, the results indicate that the mineral phases are most likely a mixture of calcite and vaterite. These data therefore correspond with the results of the XRD analyses obtained during the 96-h experiment. These data can be compared to the HRTEM results showing the interplanar distances in the possible mineral structures on the surface of the P1 phage capsids. The distances determined are approximately 315–316 nm (Fig. 8F). The closest corresponding indices (hkl) are (101) and (104) for vaterite and calcite (306 nm and 303 nm, respectively).

### Viability test after recrystallisation

The phage viability test (defined as a test of the ability to infect *E. coli* and *P. syringae* for P1 and Phi6 phages, respectively) showed that the released phages lost their ability to infect after recrystallisation. No signs of bacterial lysis were found, which was manifested by the absence of lysis on the semi-liquid agar plates (Table 1). Therefore, it can be assumed that the phages after recrystallisation may have been either damaged or mineralised to some extent, leading to loss of viability. At the initial stage, the viability test was positive, so the presence of lysate plaques on agar was observed over a wide range of dilutions of the solution after precipitation. However, the number of phages was lower than in the phage lysate used for the experiments, which should not be surprising as some of the phages were bound to particles of precipitated vaterite.

**Table 1:** Phage viability test conducted at the initial stage (directly after precipitation) and after 14 days and recrystallisation to calcite.

	Series of dilutions								
	10^−2^	10^−3^	10^−4^	10^−5^	10^−6^	10^−7^	10^−8^	10^−9^	10^−10^
Initial									
P1	L	L	L	L	L	L	L	L	NL
Phi6	L	L	L	L	L	L	L	NL	NL
After 14 days									
P1	NL	NL	NL	NL	NL	NL	NL	NL	NL
Phi6	NL	NL	NL	NL	NL	NL	NL	NL	NL

L, Presence of lysate plaques (indicator of phage viability); NL, No lysis; Series of dilutions indicate the volume (mL) of initial material used for viability test. The higher the dilution, the more phages had to be in the test material.

## Discussion

### Role of microorganisms in vaterite formation

Vaterite, as a metastable phase, transiently forms during the precipitation of calcium carbonate^6^. However, the precipitation conditions strongly influence both the shape of vaterite crystals and whether vaterite forms at all^30^. It is also known that the presence of accompanying substances during precipitation, especially organic compounds like surfactants, proteins, or other impurities, as well as bacteria, can induce vaterite formation^8,9^. This is particularly important in the context of biological mineralisation, as it has been shown that vaterite can form in the immediate surroundings of bacterial cells, as well as in the cells themselves. Thus, it is even possible to create new materials such as bacterial/vaterite composites^31^. Hence, so many practical applications are seen in biologically-induced or -influenced mineralisation processes^32^. Among the factors that may promote vaterite formation, bacterial viruses have also been listed^10^. Thus, very different possible factors need to be considered here. It has been shown that the appearance of aragonite instead of calcite can be influenced by the presence of magnesium ions in appropriate proportions^33^. This effect can be enhanced in the presence of collagen. Can the formation of vaterite also be promoted in this way? Magnesium ions play a key role in the interaction of the phage with the bacterial cell surface conditioning successful infection. One could assume that magnesium ions could always be adsorbed on phage capsids. Whether such a scenario is possible is difficult to confirm here. The presence of magnesium could induce the formation of nuclei in the form of vaterite nanoparticles and further crystallisation already with the formation of larger vaterite spheroids. But if, in fact, the presence of magnesium on phage particles were to make a difference, one would expect at least traces of aragonite. Here, it was not detected in any sample. The collagen may also affect the formation of not only metastable phases but calcite itself^34^. The presence of collagen changes the precipitated calcite from perfectly formed crystallites, to faceted rhombohedral crystals covered with surface defects. Collagen has been shown to inhibit the growth of calcite crystallites, resulting in the appearance of new surfaces. This can also be seen herein that there is inhibition of growth and the appearance of new planes in calcite. Thus, it could be hypothesized that the presence of phage capsids, which are basically larger protein particles, could act in a similar way. In addition to hypothetically stabilising vaterite, phage particles could interfere with calcite growth. The presence of proteins and various types of polymers could promote the stabilisation of vaterite^35,36^. It has also been experimentally demonstrated that the destruction of the structure of protein capsids, for example, by treatment with proteinase, does not favour vaterite formation^10^. Thus, it is possible that the structure of phage capsids, typically icosahedral with a hexagonal crystal lattice, may somehow promote vaterite formation. There is also another important aspect. The process of inducing the precipitation of vaterite can technically be carried out by many methods (mainly through the presence of chemical admixtures). However, in nature, if vaterite formation can occur somewhere, it is likely to be mainly through the presence of biological agents such as bacteria and viruses. The latter, as shown herein, can form structures containing both vaterite particles and viral capsids and, in addition, these particles can be of similar size. The implication of this is that, in contrast to vaterite forming in the presence of bacteria, a unique composite structure can be formed consisting of a large number of viral particles, possibly with a highly developed surface area. This could potentially be of considerable importance in the study of modern carbonate sedimentary environments. The experiments presented here allow to provide a conceptual model of the processes leading to the formation of such structures.

### Conceptual model of virus-induced vaterite formation

**Fig. 9:**
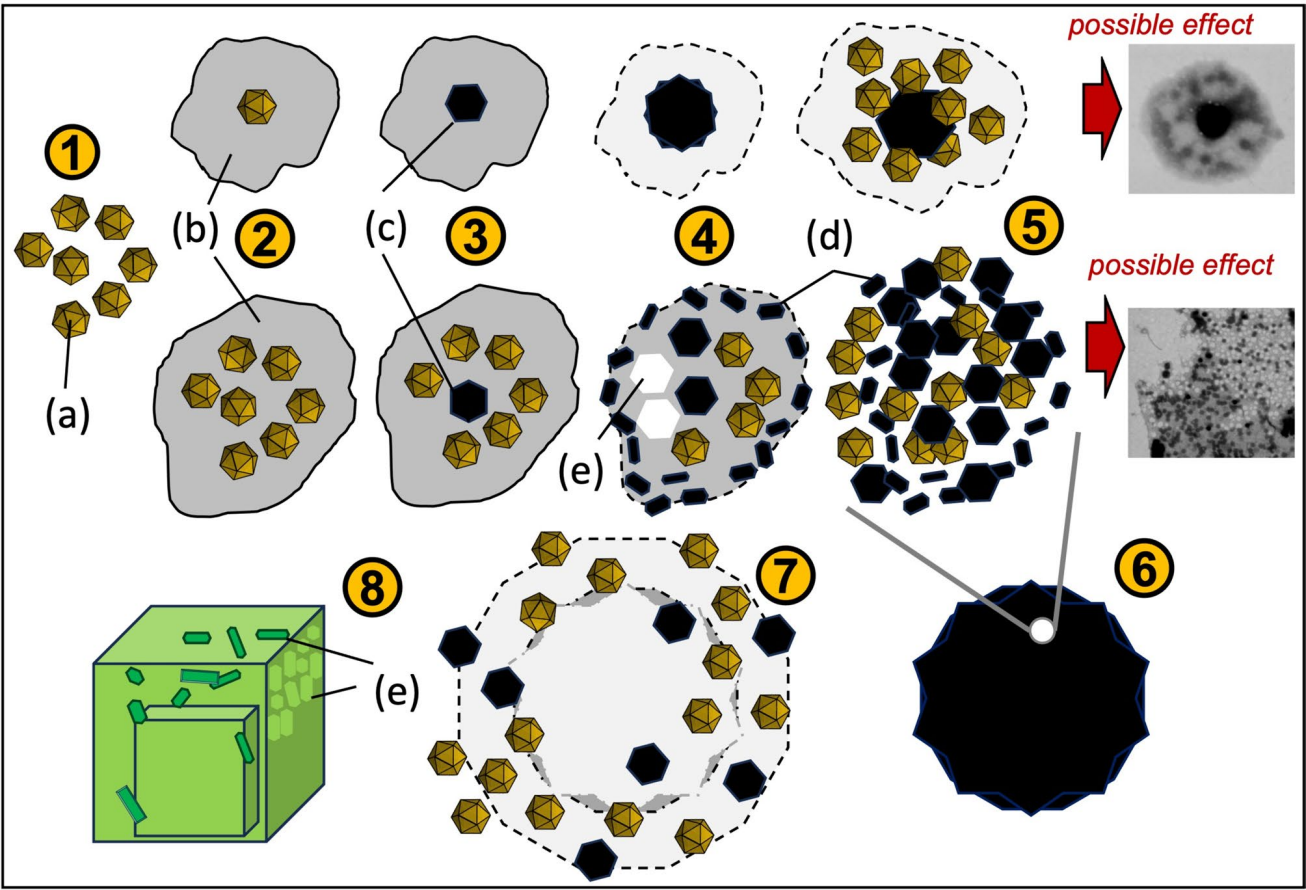
Mechanism proposed. When viruses (a) are in solution where the solubility product is exceeded, they can act as a developed precipitation surface (1). Then, ACC (b) can form directly in the viral surroundings (2). This facilitates the formation of vaterite either directly binding the viral capsid (c) or surrounded by viral particles (3). Further formation of vaterite, and possible growth of nanocrystallites (d), does not have to be directly related to the presence of viral capsids (STEM shows many smaller than capsid electron-dense mineral phases) (4). At this stage, some capsids may be released from the ACC matrix, creating clearly visible voids (e). Further aggregation of vaterite crystallites and viral particles can lead to formation of a larger spherical vaterite structure (5, 6). However, vaterite, as a metastable form, recrystallizes within several dozen hours, releasing previously bound viral particles probably at various stages of mineralisation (7). In effect, calcite crystallites are formed but with visible fine, irregular crystallites and a rough surface (e), which is different from calcite crystallites formed without the presence of viruses (8).

Undoubtedly, the presence of viral material leads to the enrichment of precipitated calcium carbonate in the form of vaterite. Previous studies, however, did not directly demonstrate how phage capsids associate with precipitated calcium carbonate. Neither did they show whether the presence of lipid envelopes of phages such as Phi6 changes anything. The data presented herein show the possible role of phages, or viruses in general, in the process of carbonate precipitation. Based on the obtained results, a hypothetical mechanism showing the role of phages in this process can be proposed (Fig. 9). It seems that phages may act either as a surface that facilitates the appearance of ACC or as nuclei undergoing organomineralisation, which should promote vaterite formation. It appears that phages can bind intra-structurally to vaterite spheroids, and not just on the surface of precipitated mineral phases. Thus, the observed fluorescence and associated anisotropy most likely came from phages bound inside the spheroidal structures. Furthermore, it should be emphasized that the presence of phage capsids affects many other processes that can lead to: (i) aggregation/agglomeration of mineral particles (important for the formation of vaterite spheroids); (ii) selective binding of phages to vaterite nanocrystals; (iii) formation of structures with a developed surface due to the formation of voids after capsids; (iv) formation of defects on the surface of calcite crystals, which may lead to high surface area. All these effects were observed during the presented experiments.

There are two concepts of spherical vaterite formation^37–40^. In both cases, bacterial viruses may play a significant role. The first concept involves the aggregation of vaterite nanocrystals. It requires the formation of a large number of small crystals, which can then form larger spherical structures. The presented studies suggest that phages could influence this process both through organomineralisation of capsids and phage aggregation. The second concept is based on the classical theory of crystal growth, where further crystal growth and the formation of a spherical form occur on the crystalline precursor. In this case, the viral capsid could act as a nucleus and the surface of the primary vaterite precipitation. The organic molecules of viruses could act as a catalyst for carbonate precipitation, as with Extracellular Polymeric Substances in bacteria^41,42^. In fact, calcium carbonate and dolomite nanospheres have been suggested to be formed at the nanometre scale by bacteria^43–45^, but this may not only be the case and as experimentally shown herein, similar spheres can be built by means of viral capsids. Therefore, the nanospheres reported by Folk^28^ may have been formed by viruses too as initially suggested by Pacton et al.^17^ and Słowakiewicz et al.^10^, what would suggest that carbonate minerals in microbially-rich systems can be precipitated by bacterial-viral communities at the nanometre scale.

### Virus-vaterite composites as a possible promising drug-delivery system

Vaterite is stable under dry conditions but undergoes decomposition and recrystallisation after approximately 48 h under wet conditions. Certainly, dry conditions are favouring the storage of vaterite, as has been shown earlier^35^. However, our experiments have also revealed that recrystallisation clearly leads to the release of viral capsids, which do not appear to associate with the calcite form. An intriguing possibility arises here for potential applications in biomedical technologies. It is known that calcium carbonate, especially vaterite, possesses important characteristics related to biocompatibility^36^. Secondly, if it can selectively bind to phages, a promising opportunity emerges to establish technologies related to both phage therapies and drug delivery. From the point of view of the application of such systems in drug delivery, the found effects of phage influence on calcium carbonate crystallisation should be very favourable. The presence of vaterite and the more developed surface of calcite crystallites should favour adsorption processes. This hypothesis should be easy to test in future laboratory studies. An additional issue concerns the ability of phages to infect bacterial cells after release during recrystallisation of vaterite to calcite. Released viruses after recrystallisation showed no ability to infect bacterial cells, and their viability seems to be destroyed after this process. However, an open question concerns the possibility of creating conditions for phages to retain such abilities after release during recrystallisation. In the present context, it is not clear whether the non-viability effects found are good or negative for possible applications of vaterite and phage-based material. This is not clear and there are no experimental data on this subject. The focus of studies in the context of phage therapies has been on the stability of phages during storage^46,47^ and the possibility of producing stable phage-containing powders by Spray Drying^48,49^. Is it possible to produce such powders based on vaterite synthesis? This remains an open question.

In conclusion, it has been demonstrated herein that bacteriophages can form structures together with mineral forms during the process of calcium carbonate precipitation. Microscopic analyses show that phage capsids can be associated with either ACC or vaterite. In the former, structures can form where capsids are somewhat immersed around a central phase, which may be crystalline calcium carbonate. In the latter, phage capsids likely induce the crystallisation of metastable vaterite and facilitate the formation of spherical vaterite forms through agglomeration. Furthermore, it seems that phages selectively bind to the vaterite phase, while probably not directly influencing calcite crystallisation. However, calcite formed in the presence of both P1 and Phi6 shows traces of numerous defects on the surface of crystallites. Recrystallisation of vaterite into calcite results in the release of phages, which are unable to infect bacteria again.

## Data Availability

All microscopic images (SEM, TEM, epifluorescence microscope) and XRD raw data are deposited in the repository (10.5281/zenodo.11159734).
